# Effects of Double-Stranded RNA Degrading Nucleases on RNAi Efficiency in Beet Moth *Spodoptera exigua* (Lepidoptera: Noctuidae)

**DOI:** 10.3390/insects16020229

**Published:** 2025-02-19

**Authors:** Guandi Wang, Qian Wang, Wenrui Liu, Jingxin Wen, Yubo Yang, Zhilong Niu, Wei Guo, Dan Zhao

**Affiliations:** 1 College of Plant Protection, Hebei Agricultural University, Baoding 071000, China; guandi991122@163.com (G.W.); wangqian_711@126.com (Q.W.); q461265042@163.com (W.L.); 15231860269@163.com (J.W.); yyb5556@126.com (Y.Y.); 13630805940@163.com (Z.N.); 2 Graduate School of Chinese Academy of Agricultural Sciences, Beijing 100081, China

**Keywords:** RNA interference, dsRNA stability, dsRNase, *Spodoptera exigua*, nanomaterial-mediated RNAi

## Abstract

The insect order Lepidoptera contains many species that are considered to be agricultural pests. Specific double-stranded RNA-degrading enzymes in some moth species decrease the efficiency of RNA interference (RNAi). RNAi refers to the efficient and specific degradation of homologous mRNA induced by highly conserved, double-stranded RNA during evolution. The dsRNase enzymes can specifically recognize exogenous dsRNA, and bind to and degrade dsRNA, resulting in the inability of dsRNA to play its role. Although dsRNases play an important role in dsRNA degradation, there has been limited research on these enzymes. In this study, we successfully identified four genes related to dsRNases (named *SeRNase1*, *SeRNase2*, *SeRNase3* and *SeRNase4*) from the genome of *Spodoptera exigua*. To overcome the rapid degradation of dsRNA in the midgut of *S. exigua*, we combined nanotechnology with biology and developed a new strategy to administer RNAi to insect pests. This binding block directed contact between the dsRNA and SeRNases to improve the efficiency of RNAi in suppressing gene expression. We demonstrate the potential of using nanotechnology to provide a novel RNAi delivery method for pest control.

## Simple Summary

RNAi is a gene suppression tool that uses double-stranded RNA to prevent specific genes from producing proteins. Unfortunately, this method does not work well on Lepidoptera pests. We successfully identified the double-stranded ribonuclease (dsRNase) gene that can degrade exogenous dsRNA in the body of *Spodoptera exigua* and constructed a novel nanodelivery system to significantly improve the RNAi efficiency against target genes. These findings demonstrated the effect of dsRNase genes on RNAi efficiency. The results reported herein contribute to the development of strategies to enhance RNAi efficiency in insects.

## 1. Introduction

*Spodoptera exigua* is a polytrophic and globally distributed lepidopteran pest known to feed on more than 170 species of plants in 35 families, including important crops such as corn, peanut, sugar beet and various cruciferous vegetables [1]. In China, *S. exigua* occurs widely, and outbreaks reported in 17 provinces have caused serious losses to agricultural production [2].

RNA interference (RNAi)—the process in which RNA molecules are involved in the sequence-specific suppression of gene expression by double-stranded RNA (dsRNA) through translational or transcriptional repression—is being increasingly used as an alternative to chemical pesticides in the control of agricultural pests [3,4]. RNAi occurs naturally in the cytoplasm of eukaryotic cells and suppresses the expression of dsRNA target genes by degrading corresponding mRNA [5]. The dsRNA can be successfully introduced into insects by injection and/or via the feed; the injection of dsRNA has also been used to screen candidate target genes under laboratory conditions, where the selected target genes must first meet the criteria of having an important role in the growth and development of pests. Once the target genes cannot be expressed normally, normal pest growth is affected. Such target genes can be used to make biopesticides for biological control [3]. Vatanparast et al. found that the molting of *Locusta migratoria* was affected after silencing the *LmNvd* gene. Lv et al. successfully screened the *Ha-AMY48*, *Ha-AMY49* and *Ha-JHE* target genes, which can be used to develop new biopesticides to control the corn earworm *Helicoverpa armigera*. The effectiveness of administering dsRNA has been verified in insects such as the migratory locust *L. migratoria* and the corn earworm *H. armigera* [6,7,8]. However, because injecting dsRNA requires the injection of each larva at a specific site, which is a time-consuming process, and because the injection amount is very low and the instrument accuracy is relatively high, it is an impractical strategy for field pest control on an agricultural scale [9]. Compared with injection, dsRNA spray is a commonly used means in RNAi, and it is both simple to operate and the administration is rapid. This is mainly achieved by spraying dsRNA on the surface of plants or feed, with pests ingesting the dsRNA present on the surface of plants or the feed when feeding [10]. While the direct spraying of dsRNA would be a better strategy to control diseases and pests, nucleases and ultraviolet light easily degrade dsRNA, and the nucleases and alkaline environment in the insect gut also affect its stability. The search for a suitable dsRNA delivery agent has been a focus of recent research on RNAi and its development [3].

One obstacle for the use of RNAi in pest control, especially in the gut and hemolymph of lepidopteran pests, is that dsRNA is very unstable and cannot induce the degradation of its own homologous mRNA [11], mainly because specific dsRNases are present in the lepidopteran gut [9]. The *BmdsRNase* protein, purified from the intestinal fluid of the silk moth *Bombyx mori*, degrades dsRNA [12], and dsRNase from the midgut of the silk moth can digest extracellular dsRNA, ssRNA and DNA [12,13]. Of the two dsRNase genes identified in the whitefly *Bemisia tabaci*, one was highly expressed in the midgut and the other throughout the body; interfering with both genes improved the RNAi efficiency of target genes [14]. In lepidopterans, dsRNases in the gut and hemolymph can degrade exogenous dsRNA; the larvae are also small, and damage from the injection is great [15,16]. Therefore, RNAi is less effective at inhibiting lepidopteran gene expression for pest control.

Nanomaterials are a class of non-viral vectors that can efficiently carry dsRNA into cells to induce the desired effects of RNAi [17]. The dsRNA–nanocarrier complexes can be efficiently and stably bound to dsRNA using one or more of these methods: electrostatic bonding, hydrogen bonding and intermolecular forces [17,18,19,20,21,22,23]. Most importantly, the use of nanomaterial-mediated RNAi can effectively protect the dsRNA from dsRNase degradation [18]. At the same time, nanocarriers can activate the clathrin-mediated endocytosis signaling pathway to help the dsRNA achieve early endosomal escape and avoid lysosomal degradation, providing maximum assurance that the dsRNA can be released within the cell to function [24,25]. Therefore, the efficient protection and delivery of dsRNA using nanocarriers can improve the efficiency of RNAi to achieve the effect of controlling the number of pests.

To identify how SeRNases affect RNAi efficiency, and to determine the factors that affect this, we examined the expression of SeRNases in different tissues and the developmental stages of *S. exigua* larvae. We developed a new nanodelivery-dsRNA system to improve the efficiency of RNAi.

## 2. Materials and Methods

### 2.1. Insects

*Spodoptera exigua* larvae were reared at 26 ± 1 °C, with a 14 h light: 10 h dark photoperiod and 65 ± 5% relative humidity in the laboratory of the College of Plant Protection, Hebei Agricultural University. Larvae were given artificial feed [26].

### 2.2. Sequence Analysis and Phylogenetic Tree Construction

The *S. exigua* genomic database was sourced from our laboratory, sequenced on BGI, but not submitted to NCBI datasets. *SeRNases* were identified in the genomic database of *S. exigua*. Amino acid sequences of the *SeRNase* genes were translated on the ExPASy website (https://web.expasy.org/translate/ (accessed on 7 October 2024)). The gene structure map used was the Exon-Intron Graphic Maker (WormWeb.org (accessed on 12 October 2024)—Exon-Intron Graphic Maker—by Nikhil Bhatla). SignalP—5.0 online software (SignalP 5.0—DTU Health Tech—Bioinformatic Services (accessed on 12 October 2024)) was used to predict the presence or absence of signal peptides. SMART (http://smart.embl-heidelberg.de/ (accessed on 12 October 2024)), SignalP 4.1 (http://www.cbs.dtu.dk/services/SignalP/ (accessed on 12 October 2024)) and Web BLAST (https://blast.ncbi.nlm.nih.gov/ (accessed on 12 October 2024)) were used to predict the domain architecture, signal peptides, and conserved regions. Phylogenetic trees of the putative dsRNases and published dsRNase sequences of 17 insect species were constructed using MEGA 11.0. The trees were visualized and colored using iTOL (http://itol.embl.de/ (accessed on 24 October 2024)).

### 2.3. RNA Extraction and Reverse-Transcription Quantitative PCR (RT-qPCR)

To study the expression of the *S. exigua SeRNases* genes in different tissues or body parts, the epidermis; fore-, mid-, and hindgut; fat body; Malpighian tubules and hemolymph were dissected from 2-day-old third instar larvae. Extraction of the hemolymph and Malpighian tubules required ten larvae at the same stage of development, and the extraction of the remaining six tissues required three larvae each. To identify the temporal expression of the *SeRNases* genes during various developmental stages, including the egg, 2-day-old first to fifth instar larvae and 2-day-old pupae and adults were used, where the insects were staged and stored at −80 °C after quick freezing in liquid nitrogen, and the total RNA was extracted using a Tiangen total RNA extraction kit (Tiangen Biotech Co., Ltd., Beijing, China). Agarose gel electrophoresis (1%) was used to verify the RNA integrity and quality. A PrimeScript FirstStrand cDNA synthesis kit (TaKaRa, Dalian, China) was used to synthesize the first strand complementary DNA (cDNA) with 1 μg total RNA following the manufacturer’s instructions. DNAMAN 6.0 software was used to design gene-specific primers (Table S1) to detect the expression levels of the *SeRNases* genes. Gene levels were normalized using the reference gene *SeActin-β*. RT-qPCR conditions were set at an initial incubation condition of 95 °C for 30 s, followed by 39 denaturation cycles of 95 °C for 5 s, annealing at 56 °C for 30 s, and extension at 72 °C for 30 s; then, the sample was processed at 95 °C for 1 min, 50 °C for 1 min, and at a temperature increase from 65 °C to 95 °C with 0.5 °C increments every 5 s. Each experimental unit contained three biological and three technical replicates. The 2^−ΔΔCT^ was used to calculate the relative expression levels.

### 2.4. dsRNA Synthesis and Preparation of the Star Polycation (SPc)–dsRNA Nanocomposites

DNA templates were amplified from cDNA using gene-specific primers containing the T7 promoter sequence at their 5′-ends, based on the nucleotides of the open-reading frame (ORF) regions of the *SeRNases* and a target gene, *SeTH*, respectively (Table S1). DsRNA was synthesized using the T7 RNA Polymerase Transcription Kit (Zhisheng Yougu Biotechnology Co., Ltd., Shanghai, China) following the manufacturer’s instructions.

Tyrosine hydroxylase (TH) is the first critical enzyme in the melanin synthesis pathway and plays an important role in pigment formation and epidermal hardening. Insect melanism is an important and special innate immune defense mechanism in insects, so we selected the TH gene as the target gene [27,28,29]. When *SeTH* is silenced, the melanin of *S. exigua* cannot be synthesized, and the color of the body surface changes, indicating RNAi efficiency. Therefore, we used *SeTH* as the target gene to observe the efficiency of RNAi at suppressing gene expression.

dsRNA and star polycation (SPc) particles were mixed at a mass ratio of 1:1 and incubated at room temperature for 30 min. To reduce electro-adsorption aggregation, ultrasonic treatment was performed for 5 min after incubation, and the mixture was vortexed promptly at high speed for 2 min to permit SPc–dsRNA generation [18]. To test the efficiency of delivery, dsRNA was loaded by SPc and assessed by gel electrophoresis; larger nanoparticles remained in gel wells and did not electrophorese. Conductive tape was applied to the sample holder, and the cover slide was cleaned with anhydrous ethanol and cut into small pieces to adhere to the sample holder without going beyond its edge. Samples (1 μL) of SPc, dsRNA and SPc–dsRNA were pumped by straw gun onto the cover glass. Samples were air-dried at room temperature for 8 h to ensure complete drying (no water residue). Morphology was observed by laser scanning confocal microscopy (Prisma E, Thermo, MA, USA) and transmission electron microscopy (JEM-1400, JEOL, Tokyo, Japan).

### 2.5. dsRNA Stability in the Midgut and Hemolymph

Midgut fluid and hemolymph were extracted using the methods from Shukla et al. [30]. Larvae (n = 30) were first anesthetized on crushed ice for 2–5 min before their midguts were carefully extracted. Extracted midguts were placed in a prechilled Eppendorf tube containing 50 µL of 1 × PBS (pH 7.6) and centrifuged at 15,000× *g* for 5 min to collect the supernatant. Midgut tissue was first pricked using sterilized pipette tips to release the gut fluids, then centrifuged at 4 °C for 15 min at 12,000× *g*. The supernatant was transferred to a new tube and stored at −80 °C until further use. The larval cuticle was punctured with sterilized tweezers and the hemolymph was collected by pipette and then transferred to a pre-cooled tube containing nuclease free phenylthiourea (PTU, 20 mg) to halt melanization. Hemocytes were removed from the hemolymph by centrifugation at 4 °C for 15 min at 12,000 × *g*; the supernatant was transferred to a new tube and stored at −80 °C until further analysis. Midgut fluid and hemolymph extracts were then separately mixed in 60 µL of ds*GFP* solution at 0.5 µg/µL and incubated at room temperature for 0, 10 and 30 min, and 1, 2, 4, 6, 12 and 24 h. Control samples contained 5 µL of nuclease-free water mixed with 30 µL of ds*GFP* solution. Following incubation, 5 µL of each sample was mixed with 2 mM EDTA to halt enzymatic reactions and then mixed with 10× loading buffer to run on a 1% agarose gel. The dsRNA integrity was visualized on the gel using a UV trans-illuminator (Clinx, Shanghai, China). The degradation assay was replicated three times from the same midgut and hemolymph extracts.

### 2.6. Release Efficiency Detection

To determine the release performance of SPc nanoparticles under different conditions, we simulated the release performance of SPc nanoparticles in insect intracellular environments (pH 6.0), midgut environments (pH 8.0) and neutral pH (pH 7.5), using PBS according to [18,19,20,21,22,23,24,25,26,27,28,29,30,31] with appropriate modifications. The SPc-ds*SeTH* complex (total ds*SeTH* 10 μg) was diluted to 10 mL for each environment at the three pH values, and incubated on a shaking table with a working power of 100 RPM and 25 °C. The 1 mL release system was removed at different time nodes, and 1 mL RNase-Free ddH_2_O was added. The dsRNA content was measured by fluorescence using the MAPADA UA-1100 Eppendorf BioSpectrometer. The SPc release curve was drawn based on the released dsRNA contents.

### 2.7. Co-Interfering Experiment of the dsSeRNases and Target Genes

To investigate the biological functions of *SeRNases* and their role in RNAi efficiency in *S. exigua*, a co-silencing experiment was performed on early third instar larvae. This developmental stage was chosen because it coincided with the highest expression of the *SeRNases* genes [2]. The combinations of ds*SeTH* with one of ds*SeRNase (1 or 2 or 3 or 4)* and ds*SeTH* with all of the four ds*SeRNases (1 and 2 and 3 and 4)* were set as the treatment groups, and the separate ds*SeTH* treatments were set as controls. After injecting 3 µL dsRNA (1000 ng/µL) into each larva (containing two or more dsRNAs and ensuring that the final concentration of each was 1000 ng/µL), each larva was fed 3 g artificial feed per replicate. In the operation of multiple dsRNA treatments, all dsRNAs were mixed and injected together to minimize mechanical damage caused by multiple injections. A total of three biological replicates were generated, each with 30 individuals. Larval body size, mortality and the presence of any abnormal phenotypes were recorded daily from 24 h after injection until sexual maturity or death. Changes in *SeTH* expression were quantitatively observed using real-time fluorescence.

For the oral delivery of dsRNA, 3 µL dsRNA (1000 ng/µL) was sprayed onto the surface of the feed (3 g) using a pipetting gun. Freshly treated feed was replaced every 24 h. A total of three biological replicates were generated, each with 30 individuals.

### 2.8. The Ability of SPc to Protect dsRNA in Larvae Measured Using Fluorescence

The ds*GFP* was labeled with Cy3; SPc-ds*GFP* (Cy3) was set as the treatment group and ds*GFP(Cy3)* was used as the control. The dsRNA for the two treatments were injected into larvae. The treated larvae were sectioned by cryostat (CM1950, Leica, Wetzlar, Germany). Each section was examined by laser confocal microscopy (LSM900, Zeiss, Oberkochen, Germany) to explore the ds*GFP* distribution throughout the larval body.

### 2.9. The Effects of RNAi on Melanin Content

Three biological repeat measurements of blackening in vivo were performed on the larvae treated with RNAi [32,33]. Third instar larvae, 48 h after ds*SeTH* treatment, were collected from each treatment, then ground and centrifuged at 4 °C with 200 μL PBS (pH 7.0) (500× *g* at 4 °C, 5 min). A total of 50 μL supernatant was transferred to a PCR tube and incubated at 30 °C for 1 h, and then 1 mM phenylthiourea (PTU) was added to terminate the reaction. Using an ultraviolet spectrophotometer (UV-1100, MADAPA, Shanghai, China) the blackness in vivo was examined by detecting the absorbance at 490 nm (A490 value) to determine changes in the amount of melanin in the moths after treatment with ds*SeTH*.

### 2.10. Statistical Analyses

Statistically significant differences were analyzed by Student’s *t* test for experiments with two samples and one-way analysis of variance (ANOVA) (SPSS, version 10.0, SPSS, Inc., Chicago, IL, USA) with the Fisher’s least significant difference (LSD) test and Duncan’s multiple range test were used for experiments with more than two samples in GraphPad Prism 9 (GraphPad Software, Inc., San Diego, CA, USA). All data are presented as mean ± standard error of the mean (SEM).

## 3. Results

### 3.1. Cloning and Sequence Analysis of the SeRNases

**Figure 1:**
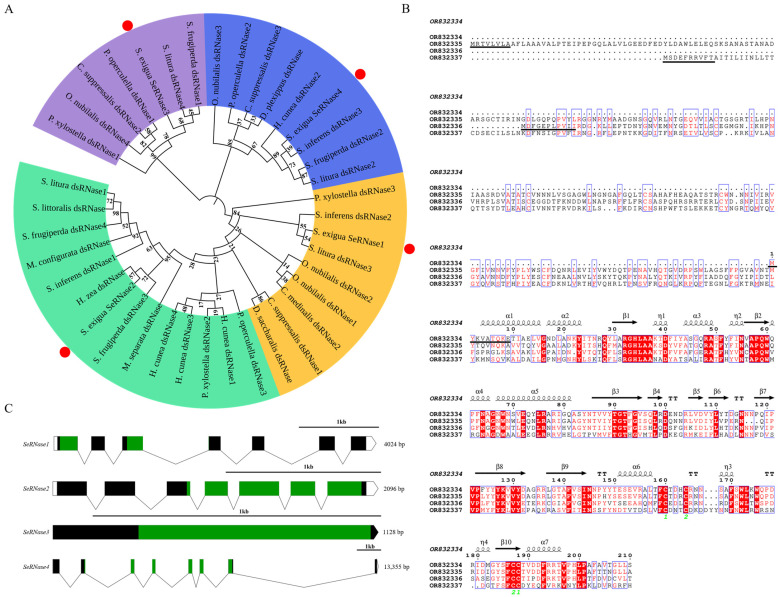
Bioinformatics analysis of the *SeRNase* genes. (**A**) Phylogenetic tree of the *SeRNases*. Red dots denote genes identified in this study. Numbers at branch nodes represent the bootstrap confidence levels of 1000 bootstrap replications obtained using MEGA 11.0. (**B**) *SeRNase* gene sequence comparison results. The conserved amino acids are shown in red boxes. The black straight line indicates the position of the signal peptide. (**C**) Genetic structure. The structure of the genome sequence is plotted to scale. Exons are represented by solid rectangles, non-coding regions are represented by hollow rectangles and the connecting line between two exons represents introns. The green areas represent functional domains.

Four *SeRNases* genes were identified from *S. exigua*. The deduced amino acid sequences contained 210–445 amino acids with molecular weights ranging between 24.10–49.33 kDa (Table 1). The predicted pI range was 5.73–9.07. Phylogenetic analysis revealed that the *SeRNases* were divided into three branches: all genes were homologous with other lepidopterans (Figure 1A). Sequence alignment and protein prediction are shown in Figure 1B; these included seven α-spirals, ten β-spirals and four η-spirals; the SeRNase protein structures were mainly β folded. *SeRNase1* and *SeRNase2* were the most similar in amino acid composition (78.04%) to each other. *SeRNase2* showed an amino acid similarity of 53.35% and 43.80% with *SeRNase3* and *SeRNase4*, respectively. *SeRNase3* showed an amino acid similarity of 44.25% with *SeRNase4*. The conserved domain of the DNA/RNA non-specific endonuclease was labeled. The signal peptide presence of the *SeRNases* was analyzed, indicating that *SeRNases* can be secreted to play a role in vitro after being synthesized by cells. The *SeRNase* gene structures are presented in Figure 1C. *SeRNase1* and *SeRNase2* contained seven exons, while *SeRNase3* and *SeRNase4* contained one and eight exons, respectively.

### 3.2. Spatial and Temporal Expression of the SeRNases

**Figure 2:**
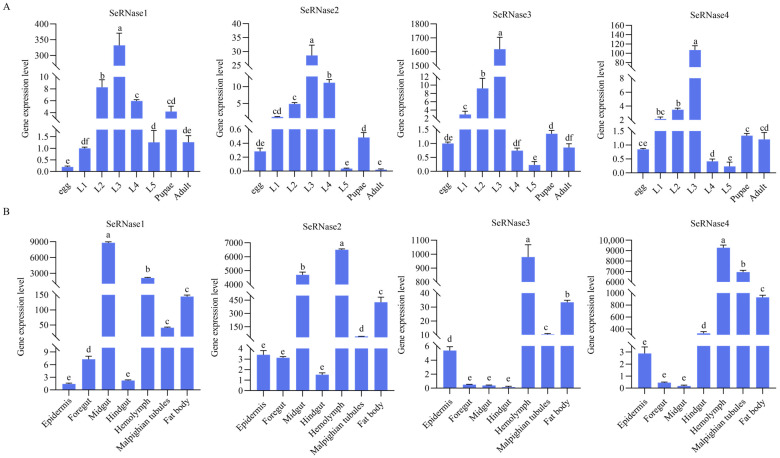
(**A**) Expression of *SeRNase1–4* in the *Spodoptera exigua* larval developmental stages. Values are mean ± SEM (n = 3). Letters above columns represent significant differences (*p* < 0.05) among samples. (**B**) Concentrations of each SeRNase in different larval tissues Values are mean ± SEM (n = 3). Letters above columns represent significant differences (*p* < 0.05) among samples.

With the *SeActin* gene serving as an internal reference, quantitative real-time polymerase chain reaction (RT-qPCR) was used to investigate the expression patterns of SeRNases throughout the larval developmental stages. SeRNase genes were expressed at every developmental stage. Notably, the expression level of SeRNases exhibited an upward trend concomitant with larval growth, peaking at stage L3, followed by a decline and ultimately reaching a relatively low level at L5 (Figure 2A).

qRT-PCR was used to analyze the expression of the SeRNase genes in L3 larval tissues on day 2. The expression levels of *SeRNase1* were most pronounced in the midgut, and low in the epidermis and hindgut. For *SeRNase2* and *SeRNase3*, expression was greatest in the hemolymph and lowest in the posterior intestine. Interestingly, the expression of *SeRNase4* was greatest in the hemolymph and lower in the mid- and foregut (Figure 2B).

### 3.3. Silenced SeRNases and Co-Silencing

**Figure 3:**
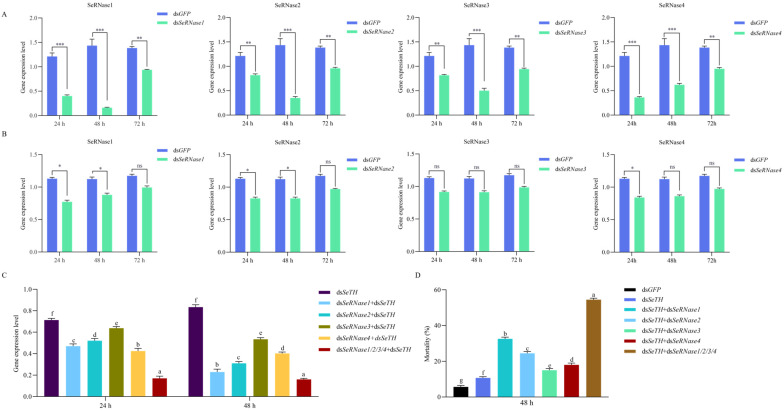
The expression levels of genes over time following the administration of RNAi. (**A**) Silencing efficiency of the SeRNases. Vertical bars represent the mean ± SEM (n = 3). Asterisks above columns indicate significant differences (** *p* < 0.01, *** *p* < 0.001). (**B**) Efficiency of the oral delivery of RNAi. Vertical bars represent the mean ± SEM (n = 3). Asterisks above columns indicate significant differences (ns indicates not significant, * *p* < 0.05). (**C**) Co-silencing efficiency. Vertical bars represent the mean ± SEM (n = 3). Letters above columns represent significant differences (*p* < 0.05) among samples. (**D**) Larval mortality at 48 h. Values are mean ± SEM (n = 3). Letters above columns represent significant differences (*p* < 0.05) among samples, as analyzed by one-way ANOVA followed by Duncan’s multiple range test.

After the L3 larvae were injected with ds*SeRNases* and fed for 24 h, *SeRNase2* and *SeRNase3* had a lower silencing efficiency than *SeRNase1* and *SeRNase4*. At 48 h, the silencing efficiency of SeRNase1 remained high (peaking at 80%). The silencing efficiency of SeRNase2 was 65%; that of SeRNase3 was 50%; and that of SeRNase4 was highest at 24 h, reaching 63%, but decreasing to 40% after 48 h. At 72 h, the expression levels of all the SeRNases gradually recovered (Figure 3A). In the oral treatment groups, the RNAi efficiency was poor: compared with controls, there was almost no silencing in the treatment groups (Figure 3B), possibly because of dsRNA degradation by dsRNases in the larval midgut, and the low intake of dsRNA.

To determine if the *SeRNases* were involved in ds*SeTH* degradation, a co-silencing injection bioassay was performed. The expression levels of *SeTH* reduced significantly over 2 d following injection with ds*SeTH* + ds*SeRNase1*, ds*SeTH* + ds*SeRNase2*, ds*SeTH* + ds*SeRNase3* and ds*SeTH* + ds*SeRNase4* compared with the ds*SeTH* control at 48 h (Figure 3C). The RNAi efficiency of each treatment group improved to varying degrees (33.4–59.7%). Injection with ds*SeTH* + ds*SeRNases (1 and 2 and 3 and 4)* increased the silencing efficiency by approximately 72.6%. When mortality was assessed after 48 h, we found that ds*SeTH* + ds*SeRNase1* was more significant than in ds*SeTH* + ds*SeRNase (2 or 3 or 4)*. Although the death rate in all the treatment groups increased, when compared with controls, the death rate of ds*SeTH* + ds*SeRNases (1 and 2 and 3 and 4)* was greater, peaking at 54.2%.

### 3.4. Preparation of the Nanomaterials and dsRNA Complexes

**Figure 4:**
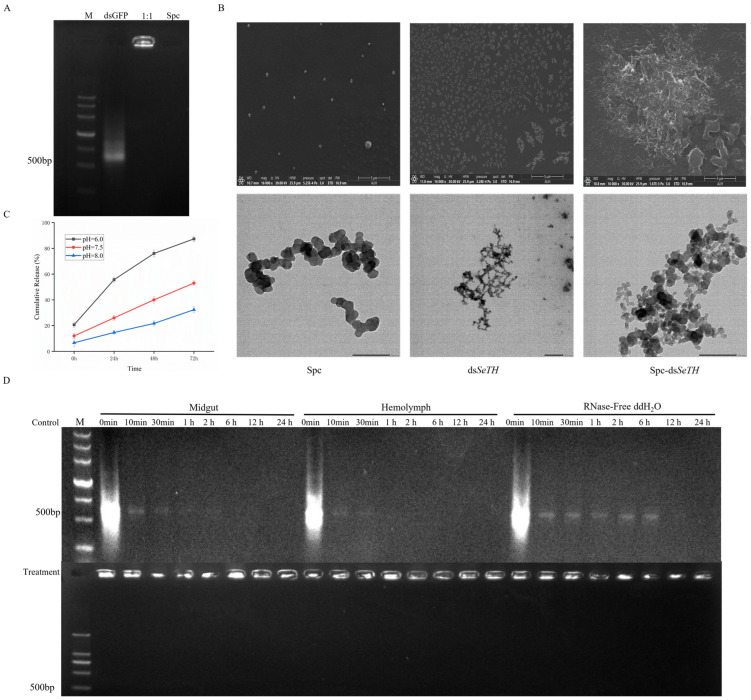
Physicochemical properties of the SPc-dsRNA. (**A**) SPc loading efficiency; dsRNA: SPc = 1:1. (**B**) SPc-dsRNA by scanning (upper) and transmission (lower) electron microscopy. (**C**) Detection of the SPc-dsRNA release efficiency. Bar graphs reflect mean ± SEM. (**D**) Protection performance of the SPc against dsRNA. The upper part of the gel diagram shows the degradation of naked ds*GFP* in vitro. The second half is the in vitro degradation of ds*GFP* coated with SPc. M = DNA Maker III.

Electrophoresis results revealed that the dsRNA was loaded by SPc and could not enter into the lane; a large amount of dsRNA remained trapped in the gel well, resulting in no lane strip (demonstrating that the SPc did successfully load with the dsRNA) (Figure 4A). With scanning electron microscopy (Figure 4B), scattered SPc particles (average size 49.52 ± 1.26 nm) and a SPc-dsRNA complex particle (average particle size 75.27 ± 1.82 nm) were apparent. With transmission electron microscopy (Figure 4B, lower), the microstructure of the SPc-dsRNA complex was visualized.

After successful construction of the SPc-dsRNA complex, the protective properties of this complex on the *S. exigua* larvae were determined. To better exert the targeted interfering ability of dsRNA, the complex must release less dsRNA in the weakly alkaline environment of the midgut and release a large amount of dsRNA within a short time in a weakly acidic cellular environment. In a simulated weakly acidic environment, the release rate of dsRNA reached 95% after 12 h, but in a simulated weakly alkaline environment, the release rate was only 20% after 48 h (Figure 4C). We measured the ds*GFP* degradation over time in the mid-intestinal fluid and hemolymph of third instar larvae; the ds*GFP* degraded relatively slowly in the mid-intestinal fluid, with low levels of the ds*GFP* detected after 1 h. In hemolymph, ds*GFP* was almost absent at 30 min. In contrast, ds*GFP* degraded most slowly in the RNase-free ddH_2_O control group, and the ds*GFP* remained after 12 h. Compared with the control group of naked ds*GFP*, the ds*GFP* did not degrade with SPc protection, and a considerable amount of ds*GFP* remained after 24 h (Figure 4D), similar to the results from Su et al., 2023 [18]. Within a short period of time, the action of dsRNA on the dsRNases in midgut fluids and hemolymph decreased to a state where it could not be detected. When protected by nanomaterials, the dsRNA persisted in large quantities.

### 3.5. Silencing Efficiency Under SPc Protection

**Figure 5:**
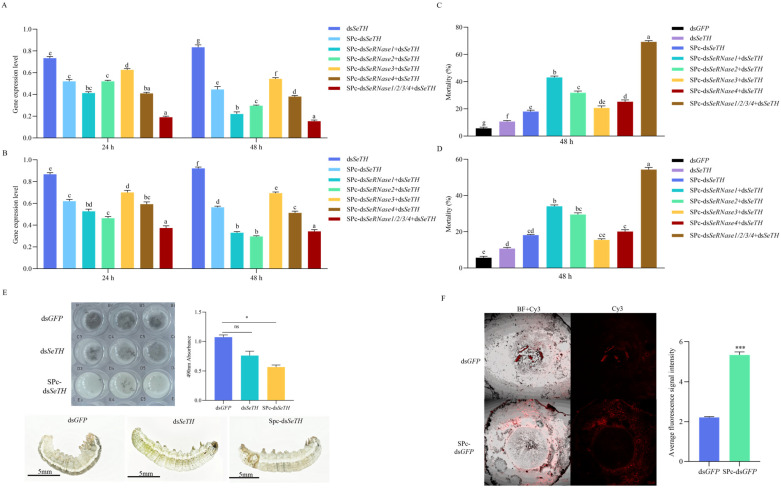
Silencing efficiency under SPc protection. (**A**) SPc increases the silencing efficiency of *SeTH*. Letters above columns represent significant differences (*p* < 0.05) among samples. (**B**) Silencing efficiency of oral delivery. Letters above columns represent significant differences (*p* < 0.05) among samples. (**C**) Larvae mortality at 48 h. Data represent mean ± SEM. Different letters indicate statistically significant differences among treatments based on one-way ANOVA followed by LSD multiple-range test (*p* < 0.05). (**D**) Larval mortality following oral dsRNA at 48 h. Data represent mean ± SEM. Different letters indicate statistically significant differences among treatments based on one-way ANOVA followed by LSD multiple-range test (*p* < 0.05). (**E**) Phenotypic changes in larvae. Significant differences between the two treatments were analyzed by an independent sample *t*-test (for the comparison of two means) (ns indicates not significant, * *p* < 0.05). (**F**) Confocal detection of the SPc protection ability, with fluorescence intensity calculated from the average of all fluorescence signals. Vertical bars represent mean ± SEM (n = 3). (*** *p* < 0.001,).

The expression of *SeTH* was significantly reduced for 2 d following injection with SPc-ds*SeTH*, ds*SeTH* + SPc-ds*SeRNase1*, ds*SeTH* + SPc-ds*SeRNase2*, ds*SeTH* + SPc-ds*SeRNase3* and ds*SeTH* + SPc-ds*SeRNase4* compared with the ds*SeTH* control. Additionally, ds*SeTH* + SPc-ds*SeRNase1–4* treatments resulted in a significantly lower expression of *SeTH* compared with ds*SeTH* + a specific *dsSeRNase (1 or 2 or 3 or 4)* (Figure 5A). Compared with the naked ds*SeTH*, the RNAi efficiency of the ds*SeTH* treatment groups wrapped with SPc was improved by 24% (24 h) and 40% (48 h). Compared with results in 3.3 without SPc, the improvement effect of SPc on the RNAi efficiency of ds*SeTH* + ds*SeRNase (1 or 2 or 3 or 4)* was 8–12%. There was almost no improvement to ds*SeTH* + ds*SeRNases (1 and 2 and 3 and 4)*. However, the lethality of the treatment group with the addition of the SPc package was significantly increased, with all increasing by >20% (Figure 5C).

The feeding treatment results followed the same trend as those of the injection group, with the RNAi efficiency decreasing by approximately 10.4–23.6% (24 h) and 8.6–17.6% (48 h) relative to injection. The mortality rate also trended downwards, and at 48 h, it was approximately 4.4–26.8% lower (Figure 5B,D). Larval skin in the treated group was light green, but dark green in controls. Similarly, larval skin in the SPc-ds*SeTH* treatments were both light green and brown in the last four segments; at this time, the silence efficiency of the ds*SeTH* alone was 18.4%, but the epidermis manifested significant changes, further indicating the sensitivity of *SeTH* as a target gene (Figure 5E). Additionally, the measurements of in vivo melanosis revealed that the melanin production of insects injected with SPc-ds*SeTH* was significantly lower than that of control insects injected with the same amount of ds*GFP* 1 h after the reaction. Although the melanin production of the larvae treated with ds*SeTH* was reduced, it was not significant compared with the ds*GFP* control group (Figure 5E). Similarly, the mortality of larvae in the SPc-ds*SeRNase (1 or 2 or 3 or 4)* + ds*SeTH* and SPc-ds*SeRNases (1 and 2 and 3 and 4)* + ds*SeTH* treatment groups increased significantly after 48 h compared with ds*GFP* in the control group (Figure 5C). Data revealed that when protected by SPc, ds*SeTH* avoided degradation by SeRNases, and the lethal effects on larvae increased.

The protective ability of SPc against ds*GFP* was better observed by labeling the ds*GFP* with Cy_3_ fluorescence. After 24 h, the molecular weight of the SPc-protected ds*GFP* exceeded that of the naked ds*GFP* in midgut sections, demonstrating the protective effect of SPc on dsRNA (Figure 5F).

## 4. Discussion

Since the first discovery of the RNAi mechanism in *Caenorhabditis elegans*, RNAi has been widely used as a tool to study gene function in various organisms, and has emerged as a new strategy for managing insect pests [34,35,36]. The use of expression modulation targeting *D. v. virgifera* protects corn roots from larval feeding damage [11,37]. However, the practical application of RNAi-based insect pest management strategies is currently challenged by the variability in the responses of different insects to exogenously applied dsRNA [11,38]. These insects either degrade dsRNA or fail to import it into the cytoplasm [39]. The dsRNA needs to persist in hemolymph or intestinal fluid for long enough to be absorbed into cells to produce an effective RNAi response [39].

To study how dsRNase reduces the efficiency of RNAi, we obtained data from *S. exigua* based on *SfdsRNases*. Four dsRNase genes were identified, named *SeRNase1–4* [40]. The endonuclease NS domain of each protein was predicted, indicating that they belonged to the DNA/RNA non-specific endonuclease family (Figure 1). Additionally, through phylogenetic analysis, these genes clustered with those of other lepidopteran dsRNase genes, further supporting that they were dsRNase homologous genes. However, the number of dsRNases in different lepidopteran taxa (insensitive to RNAi) varied from 3–6, while in most Coleoptera there were 1–4. Lepidoptera have evolved more dsRNase homologues than other insects, possibly explaining why the RNAi efficiency is lower than for other insects. In this study and others, dsRNase genes in *S. exigua* partly explains the low RNAi efficiency in Lepidoptera [40,41].

DsRNase is synthesized and secreted mainly by the midgut and hemolymph of insects [42,43,44]. In vitro experiments revealed the degradation rate of dsRNA exceeded that of the RNase-free ddH_2_O control in the midgut fluid and hemolymph (Figure 4D). The oral delivery of dsRNA may be the most viable approach to administer RNAi in pest-control applications. However, the dsRNA enzymes synthesized by Lepidoptera pests in the midgut and secreted into the intestinal lumen may degrade ingested dsRNA, reducing RNAi efficiency. A decrease in dsRNase activity in the midgut significantly increased the RNAi efficiency in *O. furnacalis* and *S. frugiperda* [45,46]. We evaluated the efficiency of RNAi in *S. exigua* by administering it orally and by injection, with the former proving to be less effective than the latter. It was also reported that RNAi efficiency was lower by injected dsRNA than oral delivery in *L. migratoria* [47]. Additionally, because dsRNA degrades rapidly before entering intestinal epithelial cells, most of the successful cases of insect RNAi have been reported via injection than by oral methods [48]. Therefore, it is more feasible to degrade the dsRNase activity or avoid direct contact between dsRNA and dsRNases to improve the RNAi efficiency.

An increasing number of studies have explored how to improve the efficiency of RNAi by delivering dsRNA via nanomaterials for pest control purposes. Many reports have confirmed that different nanomaterial–dsRNA complexes significantly improve RNAi efficiency in pests [31,49,50]. After combining SPc and dsRNA, confocal laser microscopy revealed the number of dsRNAs entering midgut cells increased significantly compared with controls. This suggested that more dsRNAs can work in combination with target sites to improve RNAi efficiency. In vitro experiments revealed the degradation rate of dsRNA in the midgut fluid and hemolymph when combined with SPc to be significantly lower, and that considerable dsRNA remained after 24 h. Release efficiency experimentation revealed that SPc-dsRNA reduced the release of dsRNA in a weakly alkaline environment, and that considerable dsRNA could be released within a short time in a weakly acidic environment. RNAi experimentation revealed the interference efficiency of the SPc-protected dsRNA treatment group to be significantly higher than that of the naked dsRNA treatment group. These experiments revealed the protective effect of SPc on dsRNA. As in previous studies, the use of nanomaterials contributed to the improvement of RNAi efficiency in pests [51,52,53]. However, the use of nanoparticles in the field has many obstacles (management and cost) [53]. Therefore, alternative strategies for dsRNase knockout for enhancing the RNAi efficiency against target genes should be considered (e.g., dsRNA-based sprays or dsRNA-expressing plants). Taking this into account, gene knockout studies have been performed using the parallel knockout of related genes transmitted by dsRNase genes using viruses.

The results that we reported for *S. exigua* are consistent with those for other Lepidoptera insects, in that dsRNA enzymes can cause dsRNA instability and lead to unstable RNAi. Additionally, through the analysis of insect dsRNase gene characteristics and functions, the prevalence of multiple dsRNases in Lepidoptera insects can be appraised and their role in RNAi efficiency evaluated. In this study, we found SPc greatly protects dsRNA from *S. exigua*, so SPc-wrapped dsRNA can improve the efficiency of RNAi. Therefore, RNAi-mediated pest-control strategies can be used in more practical applications.

## Figures and Tables

**Table 1:** Gene information analysis.

Gene	GenBank	cDNA Length (bp)	ORF (aa)	M.V. (kDa)	pI
*SeRNase1*	OR832334	633	210	24.10	8.28
*SeRNase2*	OR832335	1338	445	49.33	6.25
*SeRNase3*	OR832336	1128	375	42.75	5.73
*SeRNase4*	OR832337	1221	406	47.13	9.07

## Data Availability

All data in this study, such as the gene entry number, are available on the NCBI website (National Center for Biotechnology Information).

## Supplementary Materials

The following supporting information can be downloaded at: https://www.mdpi.com/article/10.3390/insects16020229/s1, Table S1: all primers used in this study.
