# Supplementary material for: Effects of Double-Stranded RNA Degrading Nucleases on RNAi Efficiency in Beet Moth Spodoptera exigua (Lepidoptera: Noctuidae)
Source: Insects. 2025 Feb 19;16(2):229. doi: 10.3390/insects16020229 (PMC11857036; doi:10.3390/insects16020229)
Supplement: Supplementary file 1 [file insects-16-00229-s001.zip › insects-3416635-supplementary.pdf]

Supplemental Table

Table S1 Primers used in this study

Primer	Sequence, 5'-3'	Product Size (bp)	Application
dsSeRNase1F	TAATACGACTCACTATAGGAACAATACTTGCGTGCCC	413	RNAi of SeRNase1
dsSeRNase1R	TAATACGACTCACTATAGGAAGAAGACCAGTTACGGCG		
dsSeRNase2F	TAATACGACTCACTATAGGCGCTTTACTGGTCTTGCTTC	587	RNAi of SeRNase2
dsSeRNase2R	TAATACGACTCACTATAGGGTAGTGAGGGTTGTTGATGCT		
dsSeRNase3F	TAATACGACTCACTATAGGCAACATAAAGCATCCCAACG	581	RNAi of SeRNase3
dsSeRNase3R	TAATACGACTCACTATAGGGAGTATTCCAGTTTCCGCC		
dsSeRNase4F	TAATACGACTCACTATAGGTCAAGGACATTCGCTGTAAC	543	RNAi of SeRNase4
dsSeRNase4R	TAATACGACTCACTATAGGTCCCAGTGAACACCATCA		
dsSeTHF	TAATACGACTCACTATAGGTCCTATCTTACGGTGCGGA	508	RNAi of SeTH
dsSeTHR	TAATACGACTCACTATAGGTGTGAGAACTGTGCGAAGG		
qSeRNase1F	CATCGGCCAAGCTAGTTATA	116	RT-qPCR of SeRNase1
qSeRNase1R	GATCTGTGGGTTGTTATTGC		
qSeRNase2F	TGGCAGCTTCTTCCCTGGAG	209	RT-qPCR of SeRNase2
qSeRNase2R	TGAGGAGCAGCGTTGATGAA		
qSeRNase3F	TGGAATAGCGTTTGTGGG	150	RT-qPCR of SeRNase3
qSeRNase3R	CAGAATGTGTAGCCTTCGC		
qSeRNase4F	CGATTAACTCCGAACAGTGT	230	RT-qPCR of SeRNase4
qSeRNase4R	TACTCAAGTAATGGTTGCCC		
qSeTHF	CGACAAGCCCAAACTCAT	174	RT-qPCR of SeTH
qSeTHR	CGAGCGTGATTCAATGTG		
actinF	TTCCCATCCATCGTAGGT	102	RT-qPCR of actin
actinR	GGATACCTCTCTTGCTCTGG		
